# Finasteride Enhances the Generation of Human Myeloid-Derived Suppressor Cells by Up-Regulating the COX2/PGE_2_ Pathway

**DOI:** 10.1371/journal.pone.0156549

**Published:** 2016-06-02

**Authors:** Shaoying Zhang, Kang Wu, Yufeng Liu, Yingtong Lin, Xu Zhang, Jie Zhou, Hui Zhang, Ting Pan, Yongshui Fu

**Affiliations:** 1 Institute of Human Virology, Sun Yat-sen University, Guangzhou, Guangdong, China; 2 Key Laboratory of Tropical Disease Control of Ministry of Education, Zhongshan School of Medicine, Sun Yat-sen University, Guangzhou, Guangdong, China; 3 Guangdong Engineering Research Center for Antimicrobial Agent and Immunotechnology, Sun Yat-sen University, Guangzhou, Guangdong, China; 4 Program in Immunology, Affiliated Guangzhou Women and Children’s Medical Center, Zhongshan School of Medicine, Guangzhou, Guangdong, China; 5 Guangzhou Blood Center, Guangzhou, Guangdong, China; National Cancer Institute, UNITED STATES

## Abstract

Myeloid-derived suppressor cells (MDSCs) have been known to be a key factor in the regulation of the immune system under numerous conditions such as tumors, infections, autoimmune diseases, and transplantations. In contrast to the proposed deleterious role of MDSCs in tumors and infections, MDSCs with their suppressive function are now proved to have the beneficial potential of suppressing the autoimmune response and promoting tolerance to transplantation. Therefore, the expansion of MDSCs could be a promising therapeutic strategy for many diseases. In this study, we aimed to identify FDA-approved drugs that could aid in the expansion of functional MDSCs. We performed a high-throughput screening (HTS) of FDA-approved drugs based on the *in vitro* human MDSC-differentiation system and identified finasteride (FIN) to have the best potency to aid the generation of human MDSCs. The FIN-induced MDSCs were quite similar to monocytic MDSCs with regard to their surface phenotype, morphology, immunosuppressive function, and related gene expression. Next, we aimed to determine the mechanism of action of FIN and found that FIN induced the expansion of MDSCs through up-regulation of the COX2/PGE_2_ pathway by enhancing the activity of COX2 promoter. In addition, the administration of indomethacin (IND), a COX2 inhibitor, abrogated the effect of FIN. Based on these results, we suggested that FIN could find applications in the future in the expansion of MDSCs. Further development of FIN-like compounds could be a novel strategy for generating functional MDSCs for immunosuppressive therapies in various immune disorder conditions.

## Introduction

Myeloid-derived suppressor cells (MDSCs) are identified as a heterogeneous population of multipotent progenitor cells that include granulocytes, monocytes, macrophages, and dendritic cells (DCs) precursors. MDSCs are associated with tumor vasculogenesis and mediate tumor antigen-specific T cell tolerance by expressing immunosuppressive factors such as indoleamine dioxygenase (IDO), interleukin (IL)-10, arginase, inducible nitric oxide synthase (iNOS), nitric oxide (NO), and reactive oxygen species (ROS) [1]. Over the last few years, increasing numbers of investigations have demonstrated the accumulation of MDSCs under pathological conditions such as autoimmune diseases [2–4] and transplant rejection [5, 6]. Furthermore, exogenously delivered functional MDSCs have been observed to limit autoimmune-related pathogenesis in mouse models of IBD [7], alopecia-areata [8], and type 1 diabetes [9]. The adoptive transfer of induced-MDSCs also showed the capacity of prolonging the survival of allogeneic allograft in many cases of solid organ transplantation [10–13]. Given the vital regulatory capacity of MDSCs in the immune system [14, 15], these cells are expected to be a potential candidate for the treatment of immune disorders. Therefore, the efficient *in vitro* generation and the subsequent adoptive transfer of MDSCs in some hyper-responsive conditions is of great significance [16].

It is well accepted that the generation of MDSCs is dependent on two major signaling pathways. One is predominantly responsible for the expansion of MDSCs, which can be induced by various factors such as granulocyte macrophage colony stimulating factor (GM-CSF), macrophage colony stimulating factor (M-CSF), granulocyte colony stimulating factor (G-CSF), interleukin-6 (IL-6), or vascular endothelial growth factor (VEGF) through the STAT3 and/or the STAT5 signaling pathway [17–19]. The other one is principally for the activation of MDSCs, and can be induced by various pro-inflammatory molecules such as IFN-γ, IL-1β, IL-4/IL-13, or Toll-like receptor (TLR) ligands through the STAT1, STAT6 and the NF-κB signaling pathway or via the up-regulation of cyclooxygenase 2 (COX2) [20–22]. Both of these two signals are essential for the accumulation of functional MDSCs.

COX2, as one of the cyclooxygenase isoenzymes, mediates the process of catalyzing the formation of prostaglandins and thromboxane. Except for its constitutive expression in brain and kidney, COX2 is primarily an inducible enzyme that can be activated in a variety of cells in response to cytokines, mitogens, and endotoxins [23]. The major role of COX2 involves the conversion of arachidonic acid (AA) into prostaglandin-E_2_ (PGE_2_); therefore, the level of PGE_2_ is often considered as an indicator of local cyclooxygenase activity [24, 25]. PGE_2_ is a pro-inflammatory mediator produced by cancer, stromal, and infiltrating myeloid cells and acts on G-protein-coupled receptors. COX2 is believed to be the key factor regulating the production of PGE_2_ during immune responses. However, there is a positive feedback loop between PGE_2_ and COX2, and the accumulation of PGE_2_ may induce high COX2 expression under certain conditions [26]. It has previously been reported that the COX2/PGE_2_ positive feedback loop plays an important role in the generation and activation of MDSCs [27]. Artificial manipulation of this feedback would make it possible to enhance or suppress immune responses under different clinical conditions such as in cancer, autoimmune disease, or transplant rejection [28, 29].

In order to use MDSCs for immunotherapy in immune diseases, investigators have tried various methods of expanding MDSCs in vitro to replenish the deficiencies in endogenous functional MDSC production. Multiple biologically active factors including prostaglandins, growth factors, chemokines, and cytokines have been shown to promote MDSC expansion [11, 20]. In this study, we established a high-throughput screening system for FDA-approved drugs to identify those that could aid in the generation and activation of MDSCs. The most potent drug was selected; we further went on to elucidate the mechanism by which the drug induced the expansion of MDSCs. Our study will serve as a platform for the development of therapies for autoimmune diseases and in cases of transplant rejection, and simultaneously provide a supporting for the feasibility of drug repositioning.

## Materials and Methods

### Ethics statement

This research was approved by the Ethics Review Board of Guangzhou Blood Center and the Ethics Review Board of Sun Yat-Sen University. The written informed consent was provided by study participants.

### Cell culture

HEK293T cells (Cat. No. CRL-1573)were purchased from ATCC (American Type Culture Collection) and maintained in the conditioned Dulbecco’s Modified Eagle’s medium (DMEM; Gibco, Carlsbad, CA) with 10% fetal calf serum (Gibco), 100 units/ml penicillin and 100 μg/ml streptomycin (Gibco) at 37°C and 5% CO2.

### PBMC isolation

Human peripheral blood mononuclear cells (PBMCs) were isolated from healthy volunteer donors by density gradient centrifugation through Ficoll-Hypaque (Sigma-Aldrich, St. Louis, MO), following the procedures described by our group previously [30]. Purified PBMCs were analyzed or treated immediately, and cultured at 37°C in the conditioned RPMI 1640 (Gibco) supplemented with 10% fetal bovine serum (Gibco), 2 mM L-glutamine (Gibco), 100 U/ml penicillin (Gibco), and 100 μg/ml streptomycin (Gibco) in humidified 5% CO2 incubators.

### Generation of human MDSCs *in vitro* and the subset analyses

Human PBMCs were isolated from healthy volunteer donors. Then PBMCs were seeded in 24-well plate at 1×10^6^ cells/ml in the conditioned RPMI 1640 medium supplemented with GM-CSF (20 ng/ml; Peprotech, Rocky Hill, NJ) and IL-6 (20 ng/ml; Peprotech) for 6 days. Medium was refreshed every two days. On the day 6, cells were harvested and stained with PE-Cy5-HLA-DR, PE-CD33, FITC-CD11b, PerCP-Cy5.5-CD14, or eFlour 450-CD15 (eBioscience, San Diego, CA). The cell phenotype was analyzed by flow cytometry on a flow cytometer (BD LSR Fortessa, BD Biosciences, San Jose, CA), and data were analyzed with FlowJo software package (Treestar Inc, USA). HLA-DR^–/low^CD11b^+^CD33^+^ CD14^+^ cells represented a monocytic subset and HLA-DR^–/low^CD11b^+^CD33^+^ CD15^+^ cells represented a granulocytic subset.

### Compound treatment with human PBMCs

During the generation of human MDSCs *in vitro*, different concentrations of FIN (Selleck chemicals, Houston, TX) and/or IND (Selleck chemicals), or DMSO (Sigma-Aldrich) were added since the initiation of induction. For PGE_2_ induction assay, which served as a positive control, PGE_2_ (Sigma-Aldrich) was added on the 4^th^ day. For neutralization assay, PGE_2_ neutralizing antibody (Cayman Chemical, Ann Arbor, MI) was added on the 1^st^ and 4^th^ day. Medium and cytokines were refreshed every 2–3 days. On the day 6, cells were harvested and the percentages of MDSCs were evaluated by flow cytometric analysis, based on the specific surface markers HLA-DR, CD11b, and CD33 (eBioscience).

### T-cell proliferation assay

The suppressive function of FIN-induced MDSCs was measured by their ability to inhibit the proliferation of autologous T cells, as described previously [18]. Purified CD3^+^ T cells were sorted by flow cytometry and then labeled with 1 μM 5, 6- carboxyfluorescein diacetate, succinimidyl ester (CFSE) (Molecular Probe, Eugene, OR). Cells were stimulated with human anti-CD3 antibody (R&D systems, Minneapolis, MN) at 2 μg/ml, human anti-CD28 (R&D systems) at 1 μg/ml, and IL-2 (R&D systems) at 10 ng/ml, and then cultured alone or with MDSCs. After cultured for 3 days, cells were harvested and stained with PE-CD4 and APC-CD8 antibodies (BD Biosciences). T-cell proliferation was determined by flow cytometric analysis.

### Morphology study of MDSCs

Wright-Giemsa staining was performed to assess the morphology of induced MDSCs following previous reports [18]. Briefly, freshly isolated PBMCs were prepared in parallel for comparison. Cell suspension (20 μl) with a concentration of 1×10^7^ cells/ml was smeared on the glass slides and dried for the following staining. Staining procedure was performed following the instructions of manufacturer. Observation, evaluation, and image acquisition were performed using Leica DM2500 microscope (Leica Microsystems, Deerfield, IL) connecting to an automated, digital SPOT RTke camera and SPOT Advanced Software (SPOT Diagnostic Instrument, Sterling Heights, MI).

### Flow cytometric analysis and sorting

Flow cytometric analysis and sorting were performed following previous report [30]. For staining, cells were collected and washed twice with FACS buffer (0.5% BSA in PBS), and 10^6^ cells were resuspended in 100 μl FACS buffer. Cells were stained with antibodies for 30 min on ice, then washed twice and resuspended in FACS buffer for analysis. The cell phenotype was analyzed by flow cytometry on a flow cytometer (BD LSR fortessa; BD Biosciences, San Jose, CA), and data were analyzed with FlowJo software package (Tree Star Inc, Ashland, OR). Minimum of 50,000 events within the live cells was acquired. For the flow cytometric sorting, FACSAria II cell sorter (BD Bioscience) was used. The strategy for MDSC sorting was HLA-DR^–/low^ CD11b^+^CD33^+/high^ cells from live PBMCs.

### Detection of mRNA expression

RNA from human MDSCs was extracted with Trizol reagent (Invitrogen, Carlsbad, CA) followed by DNase digestion (TURBO DNA-free; Ambion, Austin, TX) and cDNA was synthesized using an M-MLV reverse transcriptase kit (Invitrogen). QPCR was subsequently performed as described earlier [30] with gene-specific primers for each target (S1 Table).

### Cell toxicity test

PBMCs (1 × 10^5^ cells/well) were treated with different concentrations of FIN or DMSO in a 96-well-plate for 24 h. Cytotoxicity assay was performed with the Cell Counting Kit-8 (CCK-8; Dojindo Laboratories, Japan). The instructions of manufacturer were followed. OD value was recorded with a Molecular Devices microplate reader.

### ELISAs for Prostaglandin E_2_

Prostaglandin E_2_ (PGE_2_) concentration was measured by sandwich enzyme-linked immunosorbent assay (Prostaglandin E_2_ ELISA kit-Monoclonal; Cayman, Ann Arbor, MI). Culture supernatants were collected on the third and fourth day of culture. ELISA assay was performed following instructions of manufacturer. Standard curve was fitted with SoftMax Pro 6.5 (Molecular devices, San Francisco, CA).

### Western blot

PBMCs were treated with FIN and cells were harvested at different time points and lysed with lysis buffer for the detection of phosphorylation of transcription factors, phosphatase inhibitor was added into the lysis buffer. The lysate were then subjected to electrophoresis, followed by transferring onto the membrane and detection with the primary antibodies including rabbit anti-STAT1 polyclonal antibody (abcam, The United Kingdom), rabbit anti-STAT6 polyclonal antibody (ABclonal, Baltimore, MD), rabbit anti-phospho STAT1 (Tyr 701) monoclonal antibody (Cell Signaling Technology, Danvers, MA), rabbit anti-phospho STAT6 (Tyr 641) polyclonal antibody (ABclonal), rabbit anti-phospho NF-κB p65 (Ser536) monoclonal antibody (Cell Signaling Technology), rabbit anti-COX2 polyclonal antibody (R&D systems), rabbit anti-nuclear matrix protein p84 monoclonal antibody (abcam), rabbit anti-β-Actin polyclonal antibody (Proteintech) and rabbit anti-GAPDH polyclonal antibody (Proteintech).

### Plasmid construction

The 986 bp promoter fragment (from -900 to +86) of COX2 gene was generated by PCR amplification using genomic DNA from PBMCs. PCR was performed using Expand High Fidelity PCR system (Roche Molecular Diagnostics Corp.) according to the manufacturer's instructions. Nucleotides illustrated here are relative to the transcription initiation site that is +1 (GenBank accession number KR709390). The following primers were used: upstream primer, from -900, 5′-CGGGGTACC CTGCAAATTCTGGCCA-3′; downstream primer, from +86, 5′-CGCGGATCCGTCTGGCTGTGGAGCT-3′. The upstream PCR primer contained Kpn I recognition site, and downstream primer contained BamH I recognition site. The PCR products were purified from agarose gel, digested, and cloned into the pMIR-REPORT vector (Applied Biosystems, Foster City, CA).

### Transfection and dual luciferase reporter assay

Lipofectamine 2000 (Invitrogen) was used for transfection of plasmids by following manufacturer's instructions. HEK293T cells were seeded in 24-well plates at a density of 5 × 10^4^ cells/well and then 5 ng of luciferase reporter construct (FL) and 10 ng of CMV-renilla luciferase (RL) were co-transfected using 1 μl of Lipofectamine 2000 within 100 μl of Opti-MEM (Gibco). The culture was maintained for 6 h with or without FIN before harvesting the cells for dual luciferase assay at 40 h. Cells were harvested and lysed with 200 μl of passive lysis buffer (Promega, Madison, WI) for the luciferase assay as previously reported [31]. FL and RL activities were measured with the Dual-Glo luciferase assay system according to the manufacturer's instructions (Promega).

### Statistics and graphs

Statistical analyses were carried out using Prism software (GraphPad). All of the data are reported as mean ± SEM. Differences were found to be significant when P was less than 0.05 or 0.01, as indicated by single (*) or double asterisks (**) within the figures. Most graphs were produced using Prism. Flow cytometry data was processed using FlowJo (Tree Star).

## Results

### Identification of finasteride as a candidate for MDSC generation by high-throughput screening

**Fig 1:**
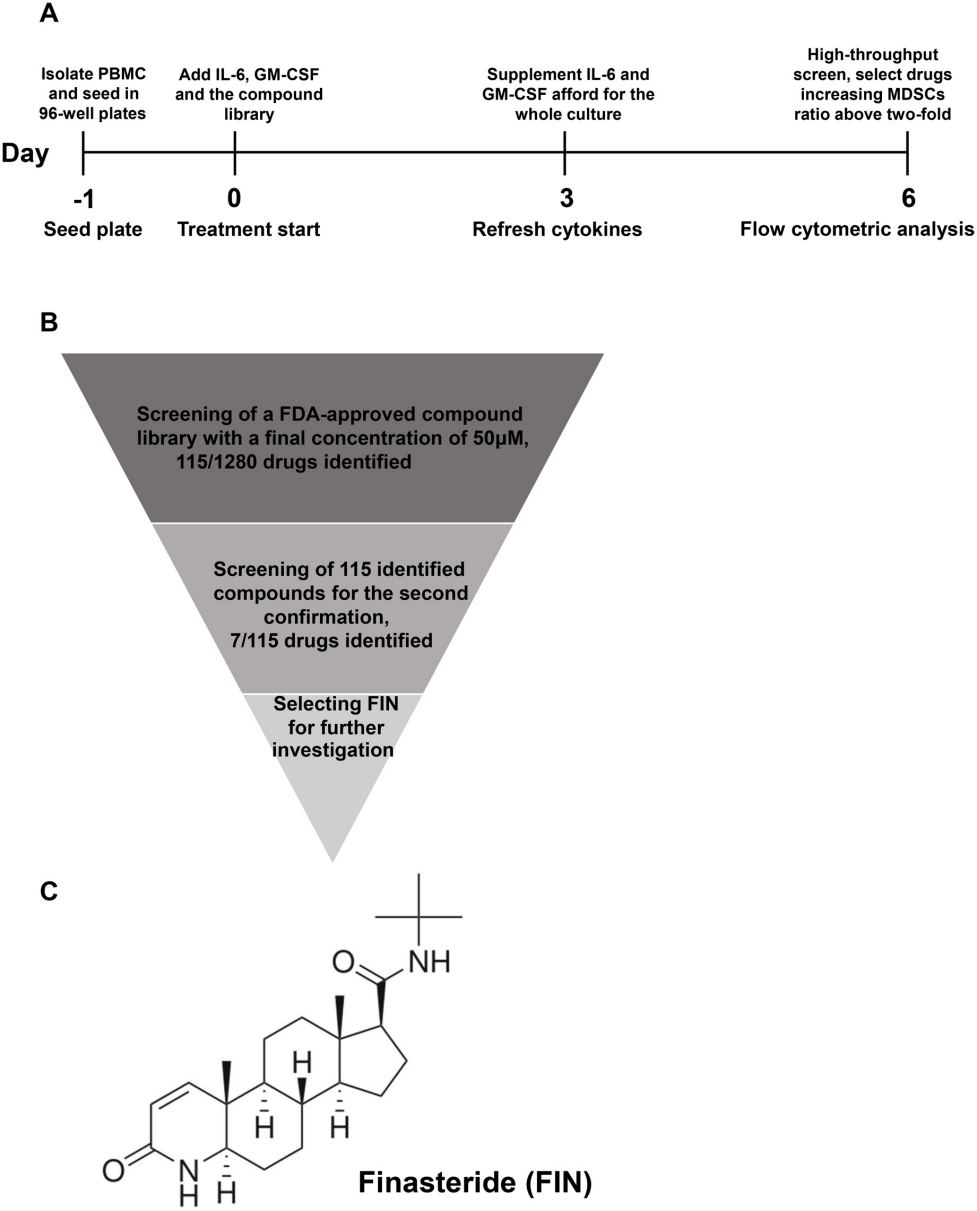
Identification of FIN by High-throughput screening of a FDA-approved drug library. **(A)** Scheme of screening with a MDSCs generation system in vitro. **(B)** Flowchart of HTS procedure. 1280 compounds from the FDA-approved drug library were screened in a dose at 50 μM for the activity on MDSCs expansion. 115 compounds that had activity (>2-fold induction) were subsequently screened for the second round in a dose at 25 μM, leading to 7 compounds that were selected. FIN is one of the best candidates and identified for further investigation. **(C)** Chemical structure of FIN.

With the aim of identifying factors that could efficiently induce MDSC expansion at a low cost, we performed a high-throughput screening of 1280 FDA-approved drugs to evaluate their active compounds. To mimic the in vivo microenvironment, a human MDSC culture system was established using fresh human peripheral blood mononuclear cells (PBMCs) in the presence of IL-6 and GM-CSF. All the 1280 compounds, each at a concentration of 50 μM, were then screened using this system in 96-well plates (Fig 1A). After 6 days of culture, the compounds that could increase the frequencies of MDSCs by 2-fold or more, in comparison to the number of DMSO-treated control cells, were selected. These compounds were then re-tested by following the same procedure, except that the concentration of the compounds in this retest was reduced to 25 μM. Seven compounds were found to be able to enhance MDSC generation sustainably even at the lower concentration. One of these compounds, finasteride (FIN), has widely been used in the clinic to treat benign prostatic hyperplasia (BPH) and has relatively less side effects; it was therefore chosen for further analysis (Fig 1B). The structure of FIN is shown in Fig 1C.

### FIN enhances the generation of functional human MDSCs *in vitro*

**Fig 2:**
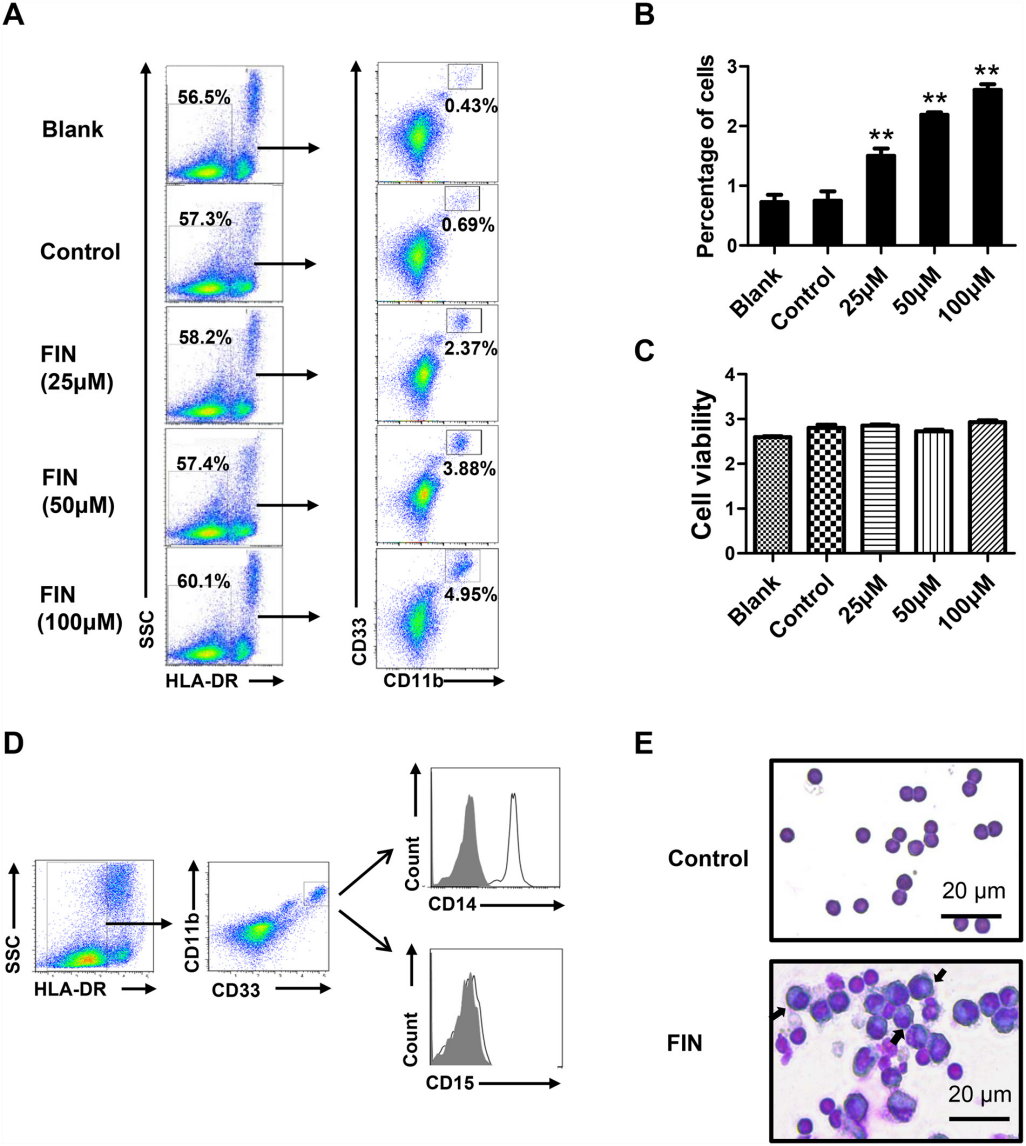
FIN enhances the generation of human MDSCs *in vitro*. PBMCs from healthy donors were cultured with GM-CSF (20 ng/ml) and IL-6 (20 ng/ml) for six days. Different concentrations of FIN were added into cell culture at day 0. PBMCs cultured with cytokines only were named “Blank”, and PBMCs treated with DMSO were used as negative control. **(A)** The proportion of MDSCs (HLA-DR^–/low^ CD11b^+^ CD33^+^) was analyzed by flow cytometry. **(B)** The percentages of MDSCs in panel A are shown in mean ± SEM from five healthy individuals. **(C)** Effect of FIN on the cell viability. Results are shown in mean ± SEM and representative of 3 independent experiments. (**D)** Expression of CD14 and CD15 was analyzed by flow cytometry to determine the FIN-induced MDSCs subset. **(E)** Wright-Giemsa staining of the FIN treated PBMCs and freshly isolated PBMCs. *p<0.05, **p<0.01, compared with the control group by student’s t-test.

**Fig 3:**
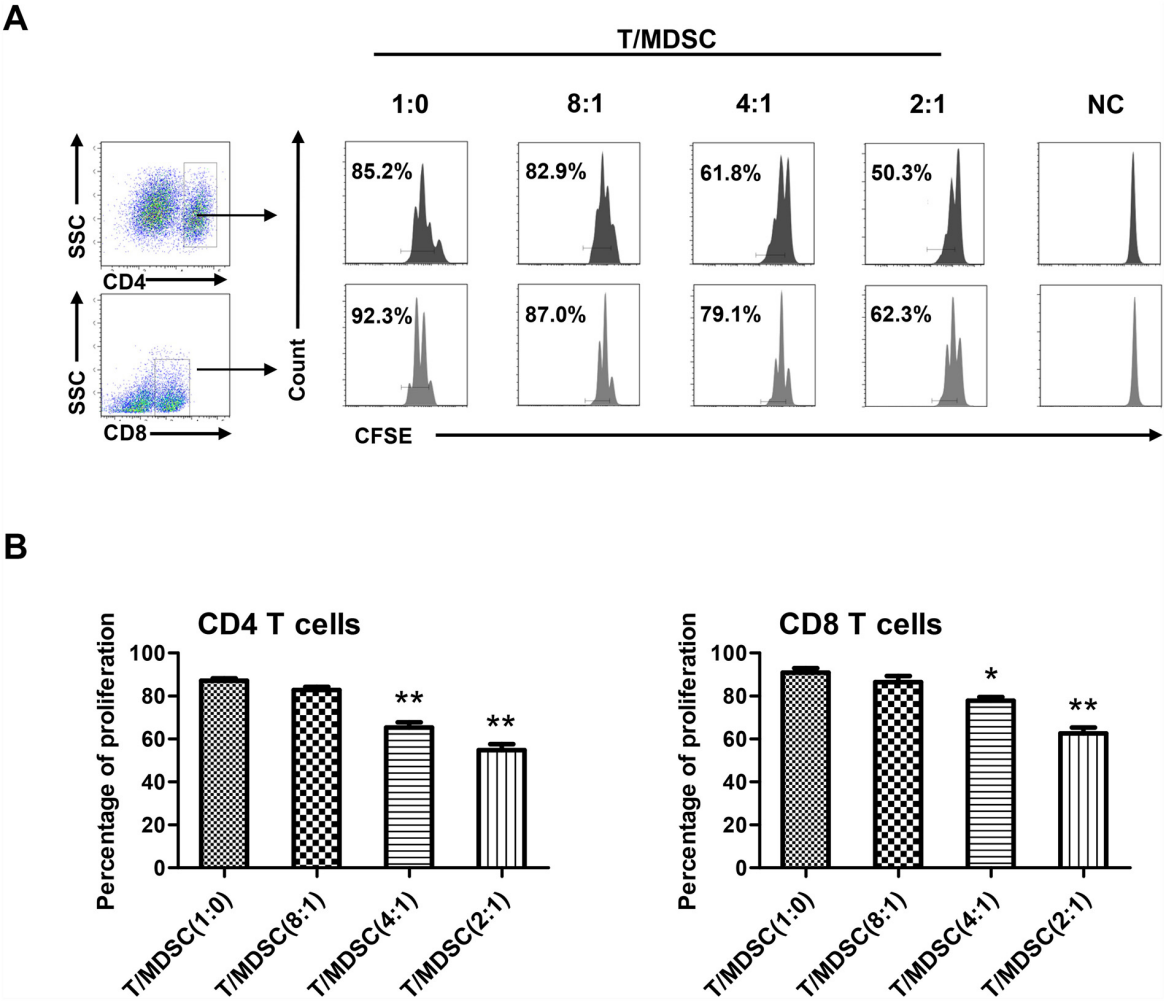
FIN-induced MDSCs suppress T cells proliferation. **(A)** Functional analysis of FIN-induced MDSCs from healthy PBMCs. FIN-induced MDSCs were sorted by flow cytometry. Autologous T cells were stimulated with anti-CD3/28 and co-cultured with MDSCs at different ratios for 3 days. T-cell proliferation was measured by CFSE dilution. Unstimulated T cells were used as negative control (NC). Results are representative of three healthy individuals. **(B)** The percentage of proliferation for CD4^+^ T cells and CD8^+^ T cells. Mean ± SEM from three independent experiments is shown. *p<0.05, **p<0.01, compared with stimulated T cells cultured alone by student’s t-test.

To further confirm the effect of FIN, dose-response analysis was performed and the results showed that FIN administration increased the proportion of MDSCs (HLA-DR^–/low^ CD11b^+^ CD33^+^ cells) in a concentration-dependent manner (Fig 2A and 2B). To exclude the possibility of FIN cytotoxicity, cell viability was measured; the cytotoxicity data (CC_50_) of FIN was found to be more than 100 μM in human PBMCs (Fig 2C). It is important to note here that, owing to their heterogeneity, human MDSCs are further classified into monocytic (M) and granulocytic (G) subsets based on the expression of specific surface markers—CD14 and CD15. We found that the FIN-induced MDSCs corresponded to monocytic MDSCs, which were CD14-positive but lacked CD15 expression (Fig 2D). Giemsa staining results indicated that the FIN-induced MDSCs had large unilobar nuclei and abundant cytoplasm (Fig 2E). As the immunosuppression towards autologous T cells is considered the major function of MDSCs, we next evaluated the effect of the FIN-induced MDSCs on T-cell responses. The MDSCs were co-cultured with autologous T-lymphocytes labeled with CFSE at different ratios. The results showed that the FIN-induced MDSCs could suppress the proliferation of both CD4^+^ and CD8^+^ T-cells in a dose-dependent manner (Fig 3).

### The COX2/PGE_2_ pathway mediates the effect of FIN on MDSC expansion

**Fig 4:**
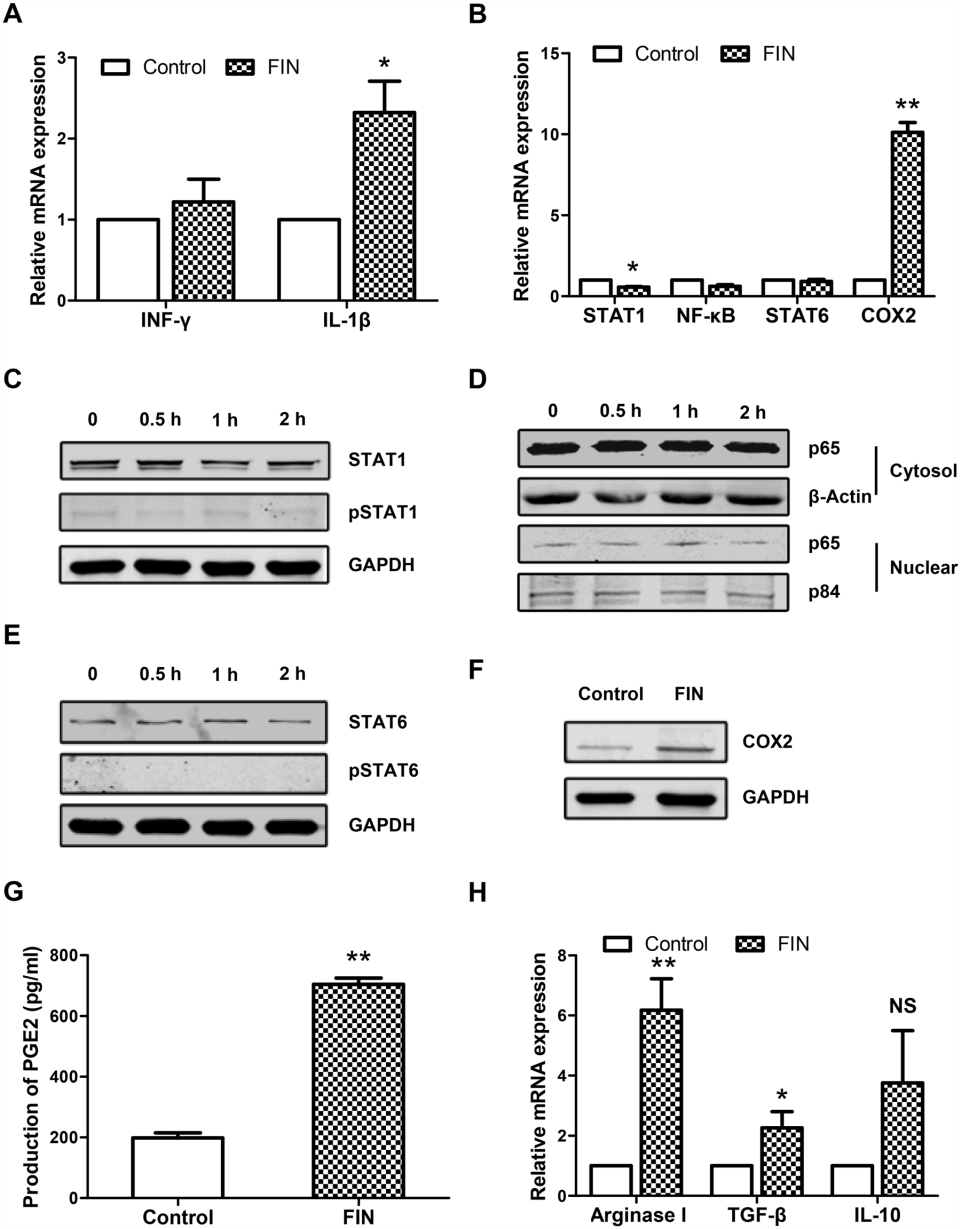
FIN up-regulates the COX2/PGE_2_ pathway. PBMCs from healthy donors were treated with FIN (100 μM) for 3–5 days. DMSO was used as negative control. Cells harvested on day 3 were for qRT-PCR, harvested at indicated time points were for western blot. **(A)** IFNγ and IL-1β mRNA expressions were determined by qRT-PCR. Results are shown in mean ± SEM of 3 independent experiments. **(B)** mRNA expressions of factors downstream of the second activating signal were determined by qRT-PCR. Results are shown in mean ± SEM of 3 independent experiments. **(C)** STAT1 and phosphorylated-STAT1 protein level was assayed by western blot analysis with cells harvested at indicated time points. **(D)** The cytosolic and nuclear extracts were analysed for RelA/p65 by Western blot analysis. **(E)** STAT6 and phosphorylated-STAT6 protein level was assayed by western blot analysis. **(F)** COX2 protein level was assayed by western blot analysis with cells harvested on day 4 and day 5. **(G)** Culture supernatant was collected on day 3 and day 4, and the PGE_2_ content was detected with ELISA. Results are shown in mean ± SEM and representative of 3 independent experiments. **(H)** mRNA expressions of factors related with suppressive function of M-MDSCs were determined by qRT-PCR. Results are shown in mean ± SEM of 3 independent experiments. **(C-F)** Results are representative of three healthy individuals. *p<0.05, **p<0.01, compared with control by student’s t-test.

We next investigated the mechanism underlying the FIN-induced generation of MDSCs. The efficacy of FIN in clinical practice is dependent on the inhibition of type II 5α-reductase. However, information from The Human Protein ATLAS online database (http://www.proteinatlas.org) suggested that type II 5α-reductase is only moderately expressed in the prostate, fallopian tubes, and liver, but that it is not expressed in PBMCs from healthy blood donors. Therefore, we speculated that there was no close relationship between the activity of type II 5α-reductase and the generation of the MDSCs, which were derived from human PBMCs cultured *in vitro*. Given that the generation of MDSCs in our study took place in the presence of sufficient IL-6 and GM-CSF, which would have afforded the first expanding signal for MDSC accumulation, we hypothesized that FIN might affect the second activating signal. To verify our hypothesis, we analyzed the mRNA expression of MDSC activation-related genes, which are mainly those encoding the inflammatory factors and members of signaling pathways. Excluding some genes such as IL-4 and IL-13 whose expression levels were too low to be detected using RT-PCR (data not shown), we found that the mRNA level of IL-1β, among the reported activating cytokines, was significantly up-regulated upon FIN administration (Fig 4A). However, among these activating signaling molecules/transcriptional factors including NF-κB, STAT1, STAT6, and COX2, COX2 solely showed a significant increase in its mRNA level upon FIN treatment (Fig 4B). When it came to the protein level, the phosphorylation of STAT1 and STAT6 were hardly detected and showed no significant alteration with or without FIN. The translocation of RelA/p65, subunit of NF-κB also showed no difference (Fig 4C–4E). In contrast, COX2 was consistently up-regulated at its protein level (Fig 4F). In previous studies, the COX2/PGE_2_ pathway had been found to play an important role in the differentiation of human MDSCs. We therefore confirmed the increased production of PGE_2_ through an ELISA assay (Fig 4G).

It is well known that the two subsets of MDSCs implement their immunosuppressive function through different pathways. G-MDSCs primarily function via ROS, while the M-MDSCs act via iNOS, arginase I (Arg I) and many other immunosuppressive cytokines [20, 32–35]. Therefore, we analyzed the mRNA expression of some key factors such as TGFβ, IL-10, Arg I, and NOS2. The expression of NOS2 was too low to be detected using RT-PCR, but all the others showed increased expression upon FIN treatment. The expression of Arg I was especially increased (Fig 4H), which was consistent with previous reports [36], indicating that COX2/PGE_2_ were the major inducers of Arg I expression in the MDSCs.

### FIN activates the COX2/PGE_2_ pathway by increasing the activity of the COX2 promoter

Due to the fact that our data showed increased mRNA expression of IL-1β (Fig 4A), which was previously reported to play a role in inducing the expression of COX2 through the mitogen activated protein kinase (MAPK) pathway [37]. It is possible that IL-1β could contribute to the up-regulation of COX2 mediated by FIN in our system. To this end, we knocked down IL-1R on the basis of FIN treatment through siRNA transfection, but found that the mRNA expression of COX2 was not significantly altered (S1 Fig). Thus there must be other mechanism(s) conducted by FIN for the induction of MDSCs.

**Fig 5:**
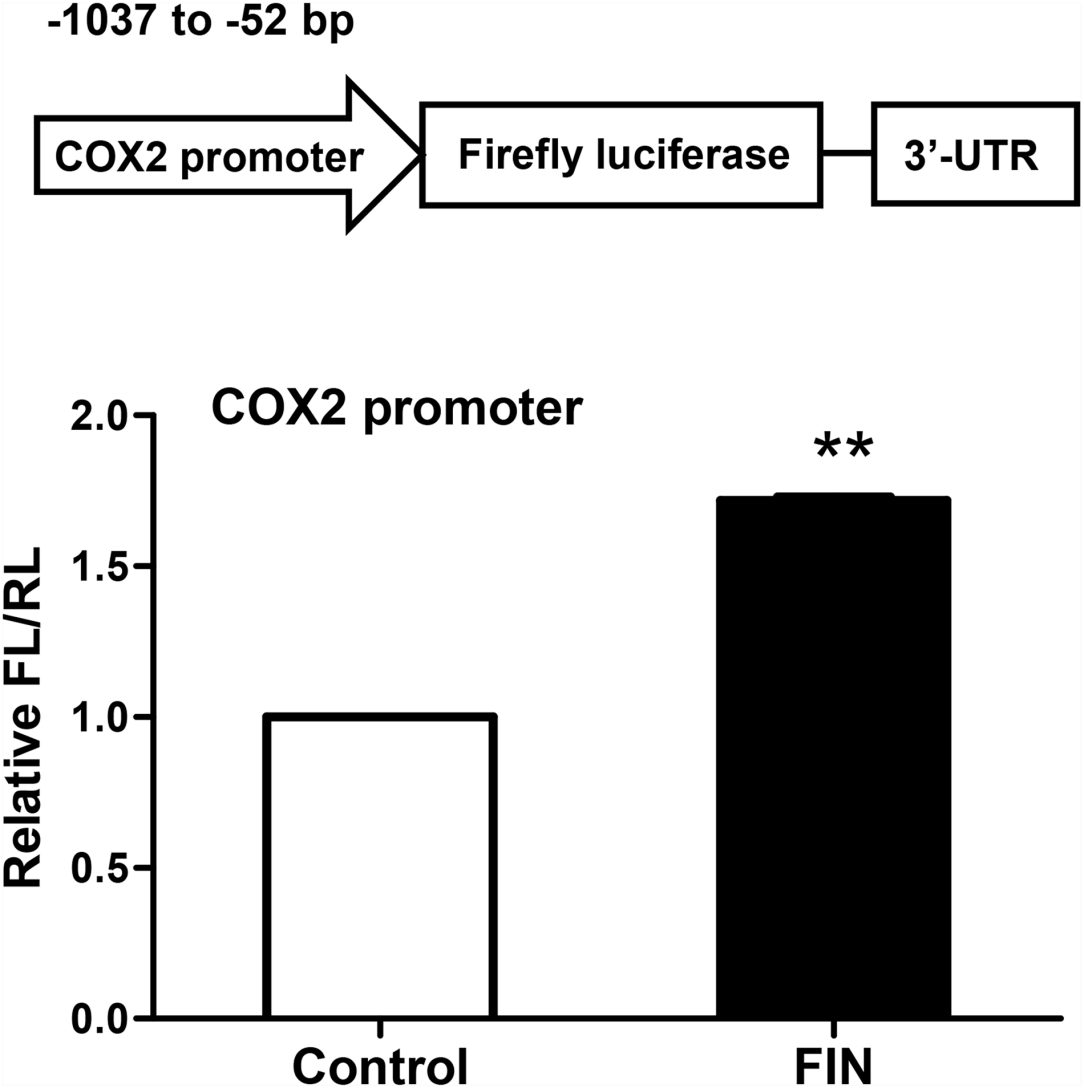
FIN mediates the COX2/PGE_2_ pathway by increasing the activity of COX2 promoter. The plasmid harboring a COX2 promoter fragment and a firefly luciferase (FL) gene (5ng/well separately) was transfected into HEK293T cells in a 24-well-plate and a CMV-Renilla luciferase (RL) plasmid (10ng/well) served as a transfection control. FIN (50μM) was treated for 6 h before harvesting the cells for dual-luciferase assay. Transfected cells treated with DMSO were set as negative control. Results are shown in mean ± SEM and representative of 3 independent experiments. **p<0.01, compared with control by student’s t-test.

While the mRNA and protein levels of COX2 were both increased by FIN treatment, we hypothesized that the increased expression of COX2 could be due to an increase in its mRNA transcription. We therefore constructed a reporter plasmid with a 986-bp fragment from position -900 bp to +86 bp of the transcription start site in the COX2 promoter region, which was followed by a firefly luciferase gene. After transfection of this reporter plasmid into HEK293T cells, the expression of the firefly luciferase gene was found to be significantly increased upon FIN treatment (Fig 5). This result indicated that FIN could enhance the activity of the COX2 promoter and thus increase the expression of COX2.

### Indomethacin (IND) abrogates the effect of FIN on MDSCs generation

**Fig 6:**
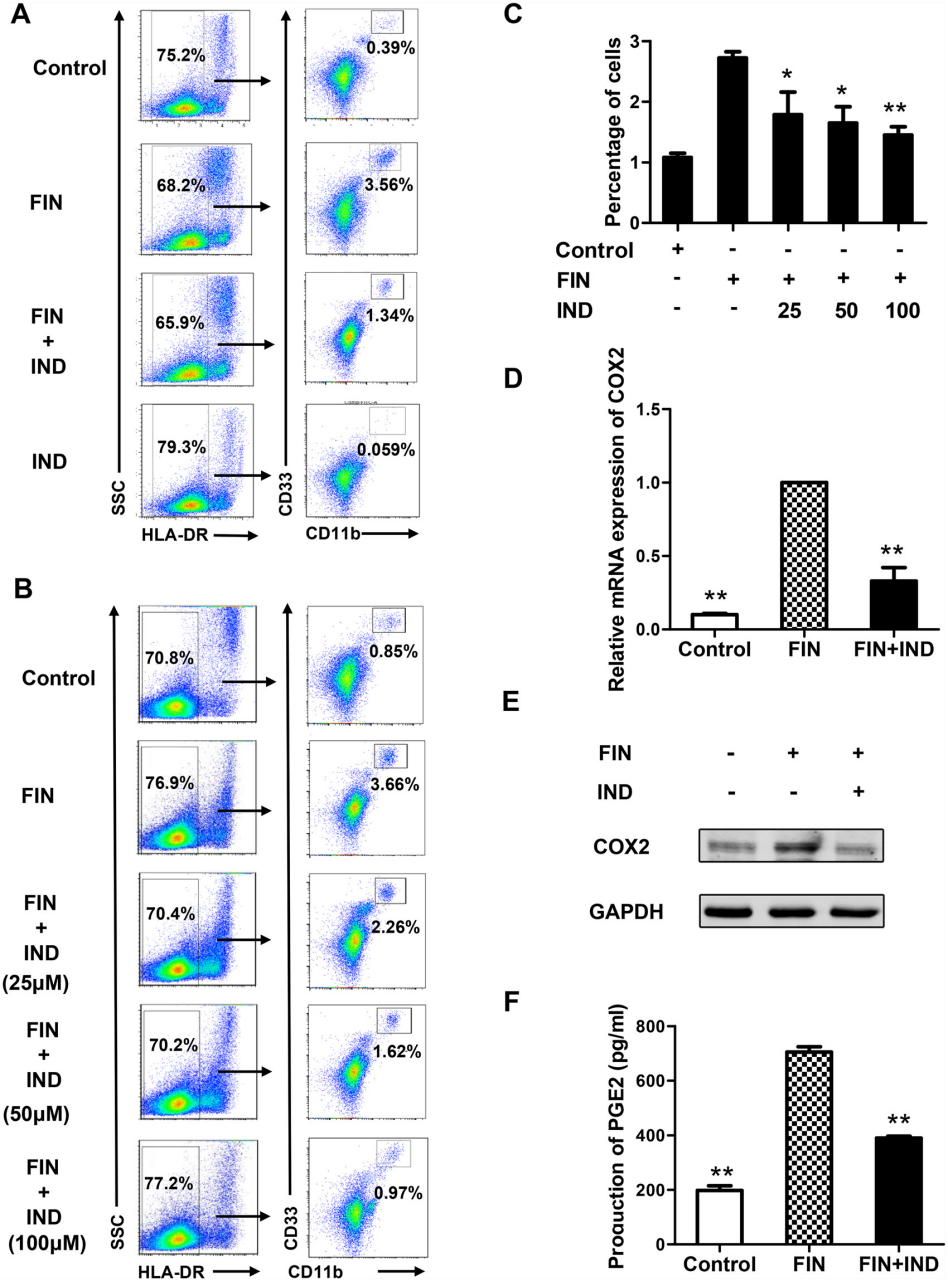
IND abrogates the effect of FIN on MDSCs generation. PBMCs from healthy donors were cultured with FIN (100 μM) and/or IND of different concentrations for six days. The proportion of MDSCs (HLA-DR^–/low^CD11b^+^CD33^+^) was evaluated by flow cytometry. **(A)** Representative result from one individual treated with 100 μM FIN and/or 100 μM indomethacin. PBMCs treated with DMSO were used as negative control. **(B)** The dose-dependent effect of IND on MDSCs expansion. **(C)** The percentage of MDSCs in panel B are shown in mean ± SEM from three healthy individuals. **(D)** The mRNA expression of COX2 gene and samples were harvested from the panel A. Results are shown in mean ± SEM and representative of 3 independent experiments. **(E)** The protein expression level of COX2 and samples were harvested from the panel A. Result is representative of 3 healthy individuals. **(F)** The production of PGE2 was detected with culture supernatant collected on day 3 and day 4 by ELISA, samples were harvested from the panel A. Results are shown in mean ± SEM and representative of 3 independent experiments. *p<0.05, **p<0.01, compared with group treated with FIN alone by student’s t-test.

To further verify the involvement of COX2/PGE_2_ pathway in the FIN-induced generation of MDSCs, indomethacin, a specific inhibitor of COX1/2 [38, 39], was added into the culture system. The results showed that the effect of FIN on MDSC generation was almost abrogated by the addition of indomethacin (Fig 6A). Furthermore, the effect of indomethacin on restraining MDSC generation was dose-dependent (Fig 6B and 6C). In addition, blocking of COX2/PGE_2_ signaling by indomethacin decreased the FIN-induced expression of COX2, as well as decreased the production of PGE_2_ (Fig 6D and 6E). Furthermore, the indicated role of PGE_2_ in FIN-mediated MDSC generation was also confirmed by the PGE_2_ neutralizing antibody treatment, which also counteracted the effect of FIN (S2 Fig). These observations supported our hypothesis that the COX2/PGE_2_ signaling pathway mediates the effects of FIN on the MDSCs.

## Discussion

**Fig 7:**
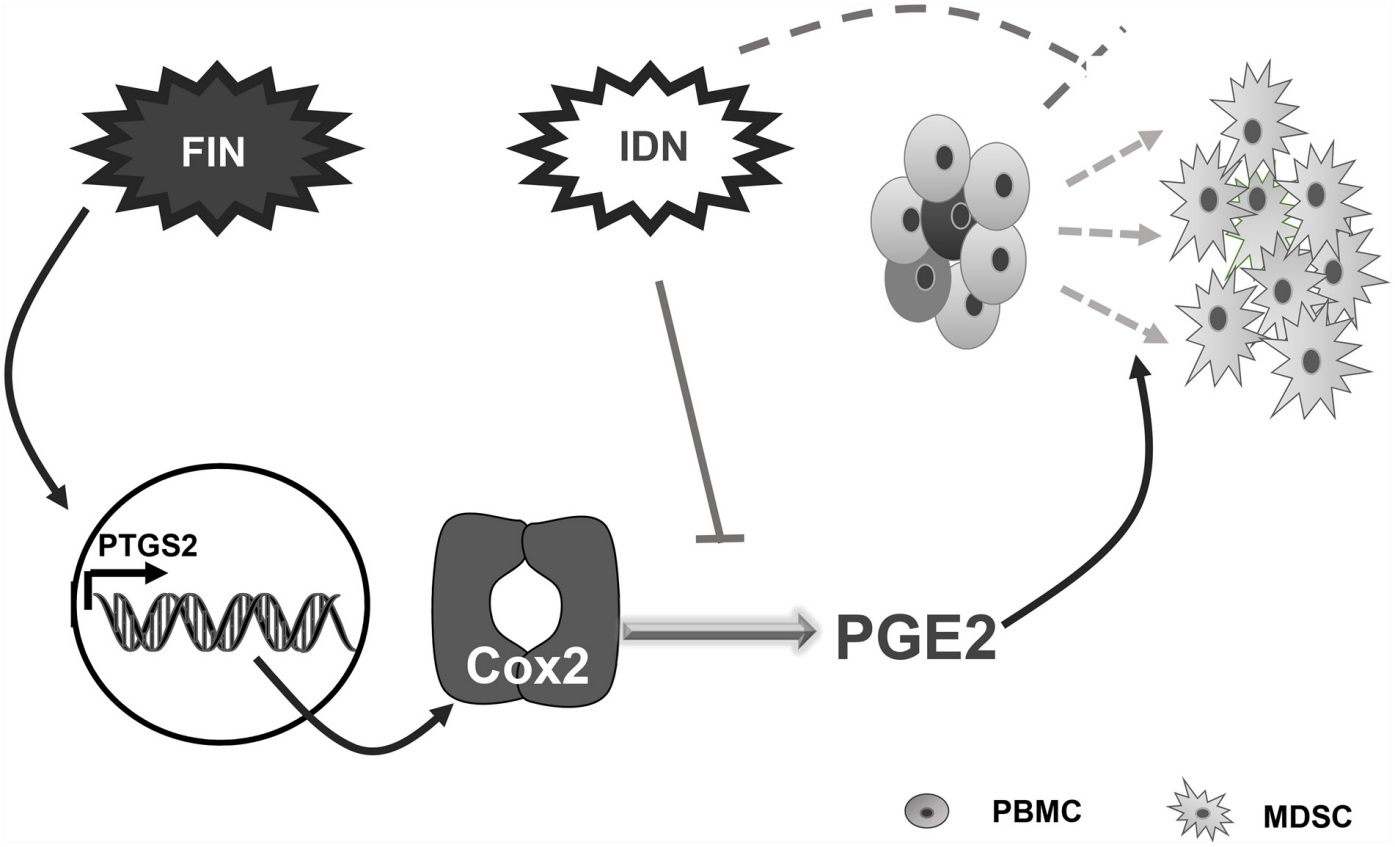
Model for the role of FIN in MDSCs generation. FIN has a role in up-regulating the expression of COX2 through increasing the activity of COX2 promoter, which leads to increased production of PGE_2_, and eventually induce MDSCs generation. During this process, the COX2 inhibitor IND could abrogate the effect of FIN.

MDSCs play defined roles in maintaining immune tolerance under a variety of physiological and pathological conditions [14, 40, 41]. In recent years, emerging reports have shown that functional MDSCs could be generated using many factors *in vitro*. However, factors available for both *in vitro* and *in vivo* usage of MDSC generation with less side effects are still needed. In light of this, in this study, we screened an accessible and reliable FDA-approved drug library, and identified FIN to be effective in generating MDSCs in a human MDSC-differentiation system *in vitro*. Since these existing drugs have already been used in humans, they have well-established dose regimen with favorable pharmacokinetics (PK) and pharmacodynamics (PD) properties as well as tolerable side effects [42], making them useful sources of application for some unintended disease conditions. FIN, as one of such existing drugs, is a potent inhibitor of 5α-reductase, which is an enzyme that converts testosterone to dihydrotesterone (DHT), and is involved in bile acid biosynthesis, androgen and estrogen metabolism, as well as in prostate cancer [43]. By inhibiting 5α-reductase, FIN prevents the conversion of testosterone to DHT through type II and III isoenzymes, resulting in a down-regulation of DHT in the serum [44]. As there is no 5α-reductase expression in PBMCs (http://www.proteinatlas.org/ENSG00000277893-SRD5A2/cell), with which we used to initiate the MDSC-inducing system, it is unlikely that FIN would induce the generation of MDSCs through the regulation of 5α-reductase. Our data indicated that both the surface phenotype and morphology of the FIN-induced MDSCs were similar to those of monocytic MDSCs. Indomethacin potently inhibited the FIN-induced generation of the MDSCs, strongly supporting our hypothesis that the COX2/PGE_2_ pathway played an important role in FIN-induced MDSC generation (Fig 7).

Conversely, although the treatment of BPH with FIN is a developed protocol in clinical practice, the FDA recently concluded that FIN administration may accompany an increasing risk of high-grade prostate cancer (http://www.fda.gov/Drugs/DrugSafety/ucm258314) and breast cancer [45]. However, the exact reason why the treatment of FIN resulted in such increased risk among 5α-reductase inhibitor-treated patients remained ambiguous. The MDSC-inducing capacity of FIN revealed in our study may provide a reasonable explanation for such increased risks. Upon administration, these FIN-induced MDSCs could induce tumor antigen-specific T-cell tolerance, thus allowing the deteriorating tumor to evade immunosurveillance. Coincidently, a recent clinical trial showed an absolute advantage in alleviating BPH when adopting a combination therapy with a COX2 inhibitor and FIN [46], which further supports our hypothesis. However, direct clinical evidence is certainly required to further verify our hypothesis in the future.

Our study suggested that MDSC induction could play an important role in the adverse effects observed in FIN-treated BPH patients. Our findings also likely provide a new perspective on the clinical use of FIN, and reveal a mechanism that may be responsible for the “evasion” of cancers that develop upon FIN administration. Our study also provides a novel insight into the advantages of rational administration and combination therapy in diseases. Besides their recognized role in cancers, MDSCs are also being gradually accepted as regulators of the immune system. Immature myeloid cells (IMCs) in naive mice are an intrinsic part of normal hematopoiesis and are not immunosuppressive when they are in an inactivated state. Upon encountering some acute stress, the IMC population will quickly undergo a transition into the activated state [14]. However, this transient IMC population with the suppressive functions of MDSCs is always short lived and thus has minimal impact on immune response. There has also reports indicating that the dysfunction of MDSC *in vivo* may be a factor driving autoimmune inflammatory pathogenesis [2]. Therefore, the administration of FIN might help to activate the dysfunctional MDSCs, initiating such immunosuppressive functions and making the regulatory network controllable; it could therefore be a novel method that can be used for therapeutic purposes in a variety of clinical settings.

In summary, in our study, we identified FIN as a compound that capable of inducing MDSC generation. We also elucidated that FIN performs this role through the COX2/PGE2 pathway by increasing COX2 expression through enhancing its promoter activity. Our findings provide a possible explanation for the increased risk of high-grade prostate cancer and breast cancer among FIN-treated patients. Further, as MDSCs play a role in the regulation of the immune system, FIN may have potential applications in the treatment for autoimmune diseases and in cases such as transplant rejection. Further studies to validate the use of FIN in such scenarios are required.

## Supporting Information

S1 Fig IL-1β is not responsible for FIN-mediated up-regulation of COX2. PBMCs from healthy donors were transfected with siIL-1R (30 nM) or siNC (30 nM) for 24 h before adding FIN (100 μM). Cells then treated with FIN for 36–48 h were harvested for RNA extraction and following qRT-PCR. **(A)** Knock-down efficiency of siIL-1R was determined by qRT-PCR. Results are shown in mean ± SEM of 3 independent experiments. **(B)** mRNA expression of COX2 upon the knock-down of IL-1R was determined by qRT-PCR. Results are shown in mean ± SEM of 3 independent experiments. **p<0.01, student’s t-test.
(TIF)

S2 Fig Effect of PGE2 and PGE2 neutralizing antibody on MDSCs generation. PBMCs from healthy donors were cultured with PGE2 alone or with PGE2 neutralizing antibody in the presence of FIN (100 μM). Cells treated with FIN (50 μM) alone were used as control; treated with DMSO were used as negative control. **(A)** The proportion of MDSCs was analyzed by flow cytometry. **(B)** The percentages of MDSCs in panel A are shown in mean ± SEM from three healthy individuals. Here PGE_2_ is treated at a concentration of 10 μM, and PGE_2_ neutralizing antibody is at 10 μg/ml. **p<0.01, compared with group treated with FIN alone by student’s t-test.
(TIF)

S1 File The individual data points behind means, medians and variance measures. (XLSX)

S2 File The original uncropped and unadjusted blots. (PDF)

S1 Table The gene specific primers for each target genes. (DOCX)
