# Supplementary figures and images for: Finasteride Enhances the Generation of Human Myeloid-Derived Suppressor Cells by Up-Regulating the COX2/PGE2 Pathway
Source: PLoS One. 2016 Jun 2;11(6):e0156549. doi: 10.1371/journal.pone.0156549 (PMC4890941; doi:10.1371/journal.pone.0156549)

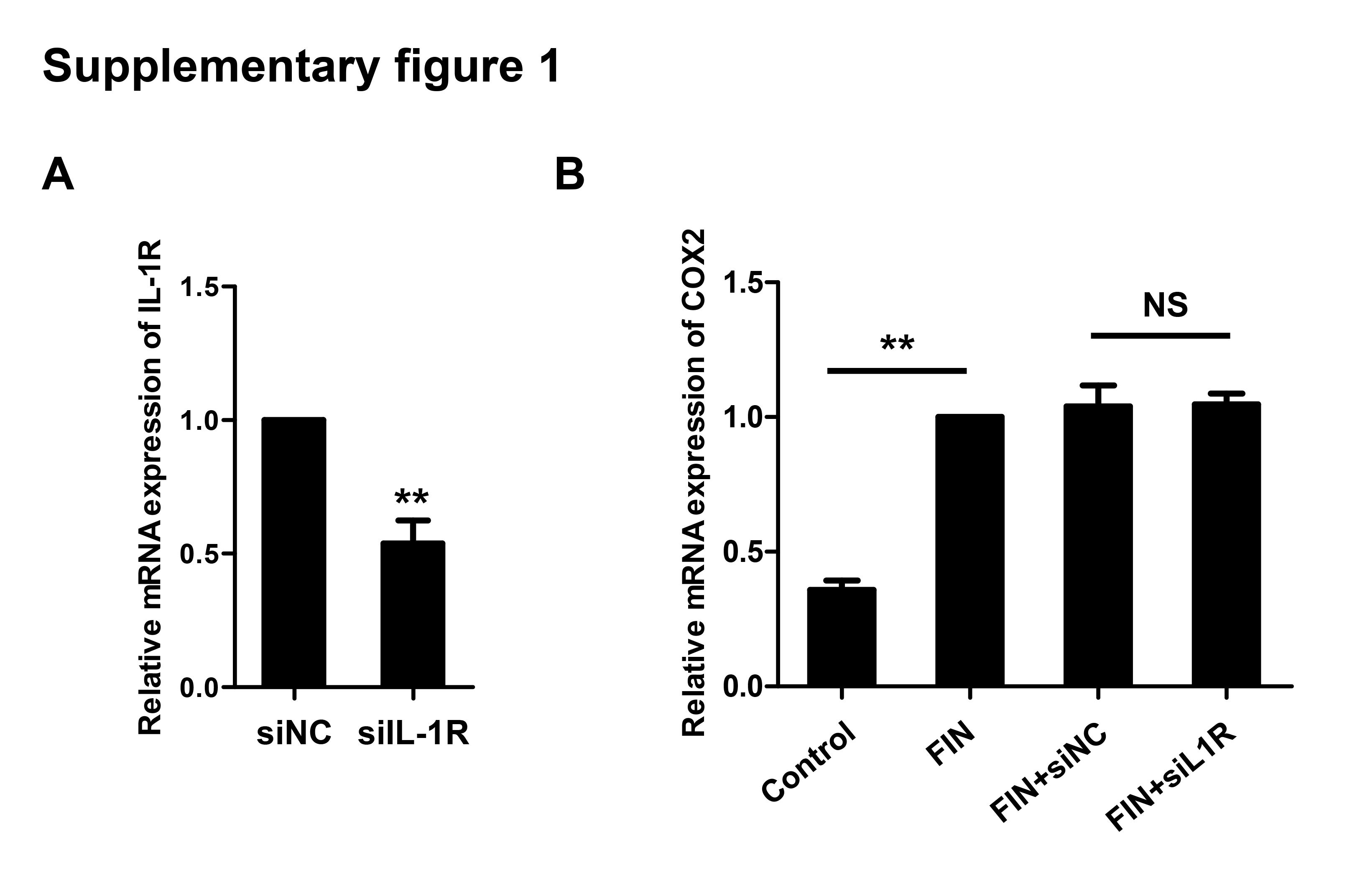

Supplement: S1 Fig — PBMCs from healthy donors were transfected with siIL-1R (30 nM) or siNC (30 nM) for 24 h before adding FIN (100 μM). Cells then treated with FIN for 36–48 h were harvested for RNA extraction and following qRT-PCR. (A) Knock-down efficiency of siIL-1R was determined by qRT-PCR. Results are shown in mean ± SEM of 3 independent experiments. (B) mRNA expression of COX2 upon the knock-down of IL-1R was determined by qRT-PCR. Results are shown in mean ± SEM of 3 independent experiments. **p<0.01, student’s t-test. (TIF) [file pone.0156549.s001.tif]

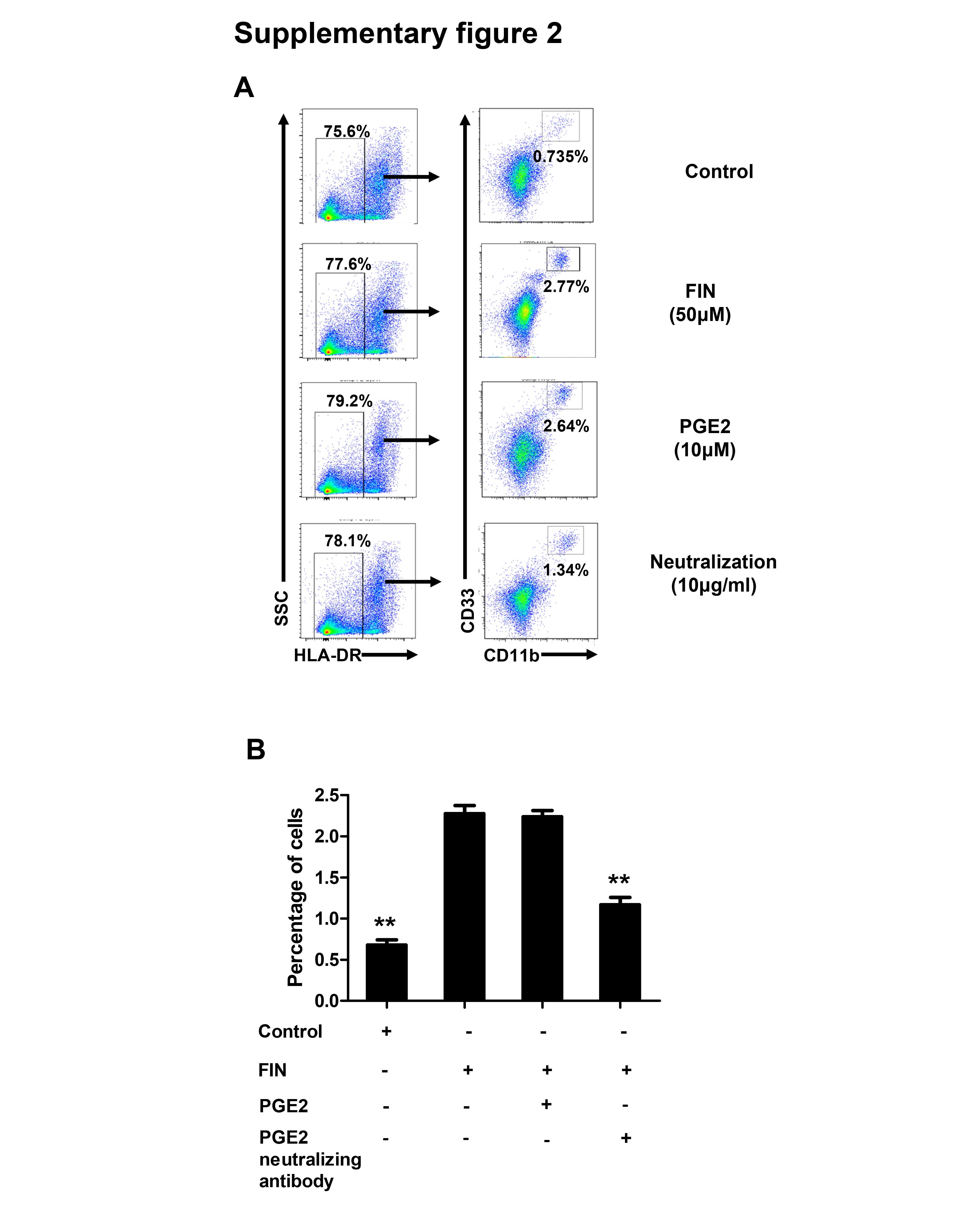

Supplement: S2 Fig — PBMCs from healthy donors were cultured with PGE2 alone or with PGE2 neutralizing antibody in the presence of FIN (100 μM). Cells treated with FIN (50 μM) alone were used as control; treated with DMSO were used as negative control. (A) The proportion of MDSCs was analyzed by flow cytometry. (B) The percentages of MDSCs in panel A are shown in mean ± SEM from three healthy individuals. Here PGE2 is treated at a concentration of 10 μM, and PGE2 neutralizing antibody is at 10 μg/ml. **p<0.01, compared with group treated with FIN alone by student’s t-test. (TIF) [file pone.0156549.s002.tif]
