# Supplementary material for: Finasteride Enhances the Generation of Human Myeloid-Derived Suppressor Cells by Up-Regulating the COX2/PGE2 Pathway
Source: PLoS One. 2016 Jun 2;11(6):e0156549. doi: 10.1371/journal.pone.0156549 (PMC4890941; doi:10.1371/journal.pone.0156549)

Original uncropped and unadjusted blots for STAT1 in Fig. 4C

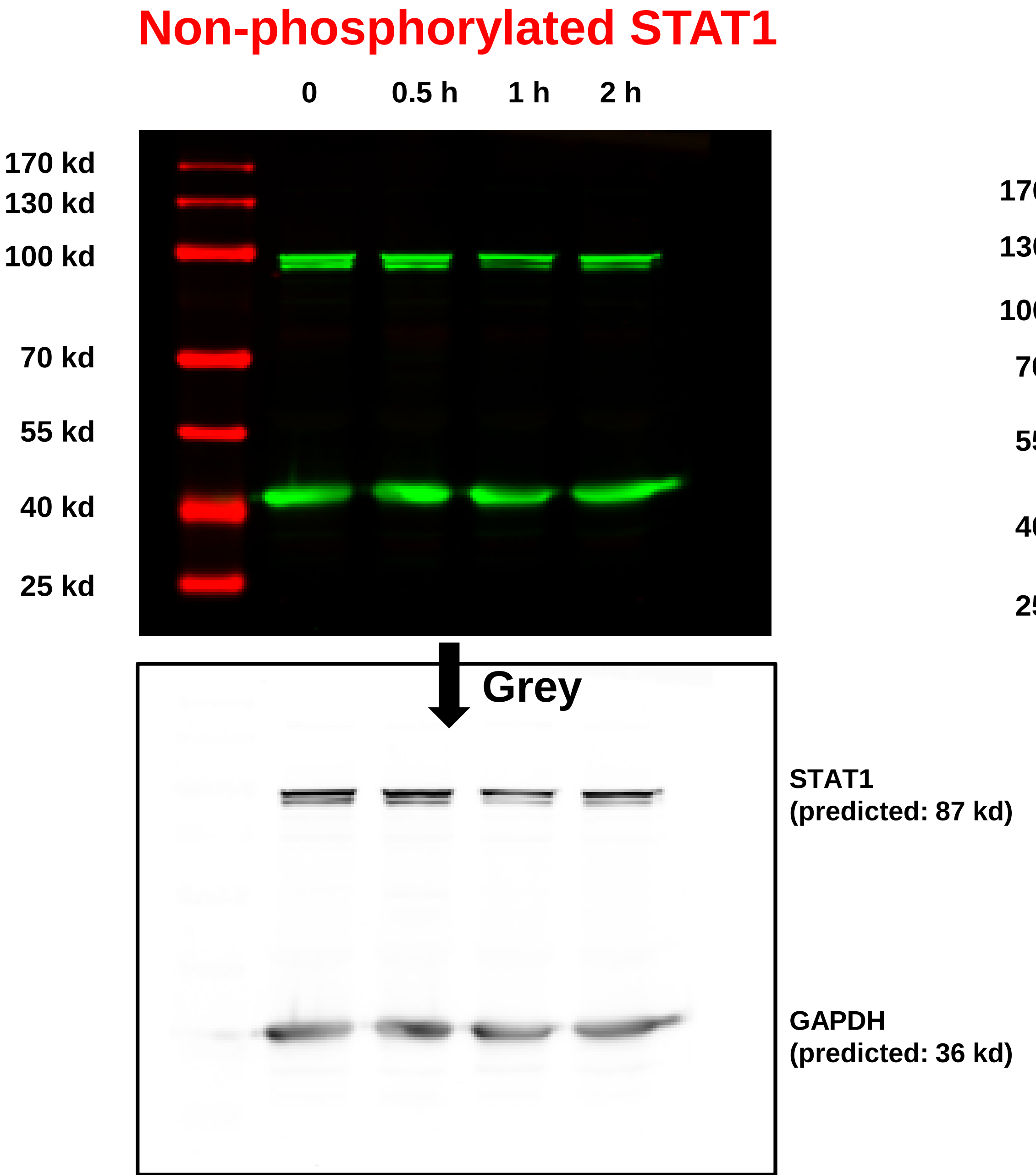

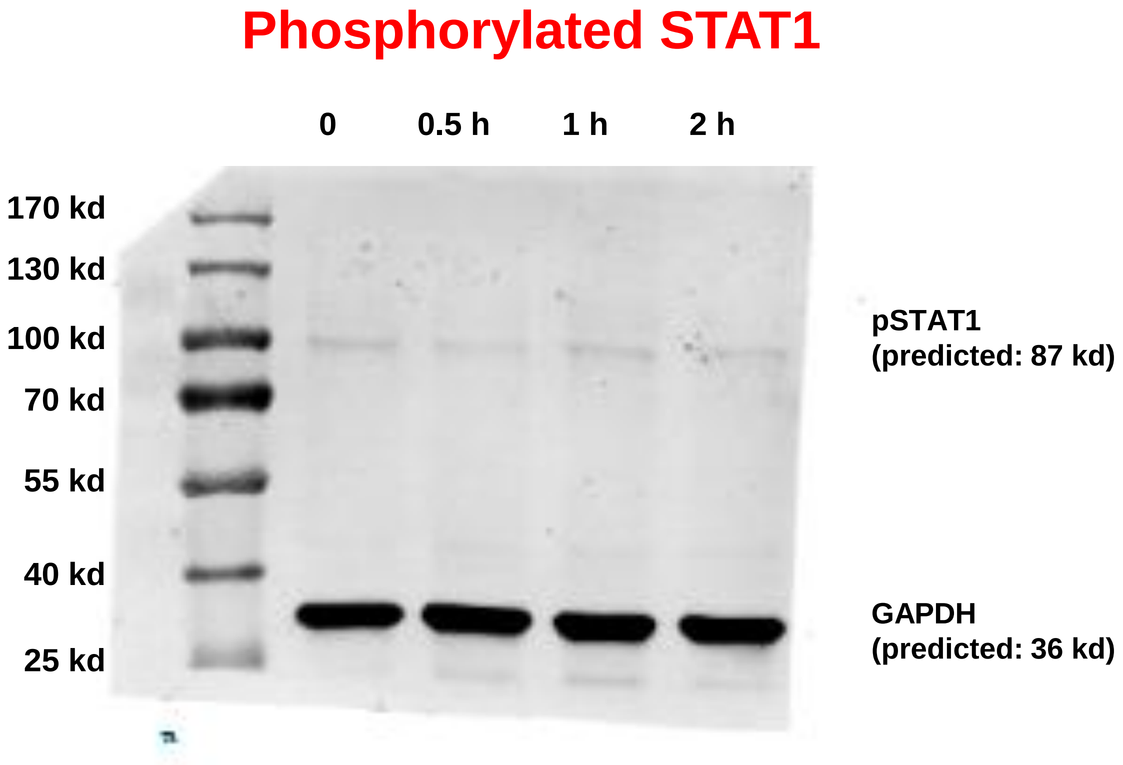

The bands for STAT1 and pSTAT1 are too close to each other. Here the same samples are presented on two gels and incubated with separated antibodies.

Original uncropped and unadjusted blots for p65 in Fig. 4D

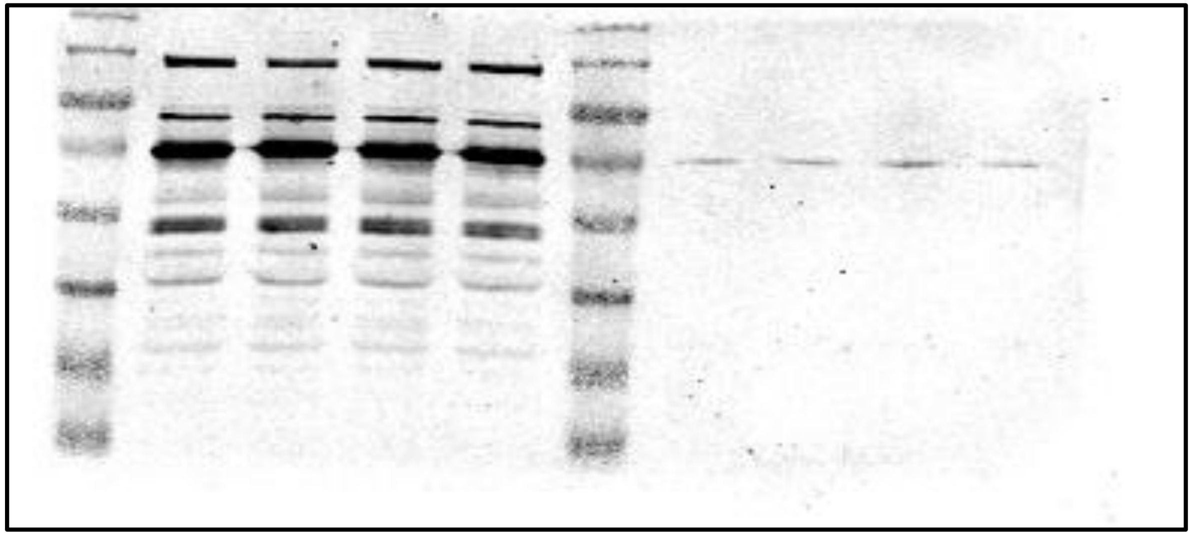

RelA/p65
(predicted: 64 kd)

The cytosol and nuclear sections are incubated with respective control antibodies.

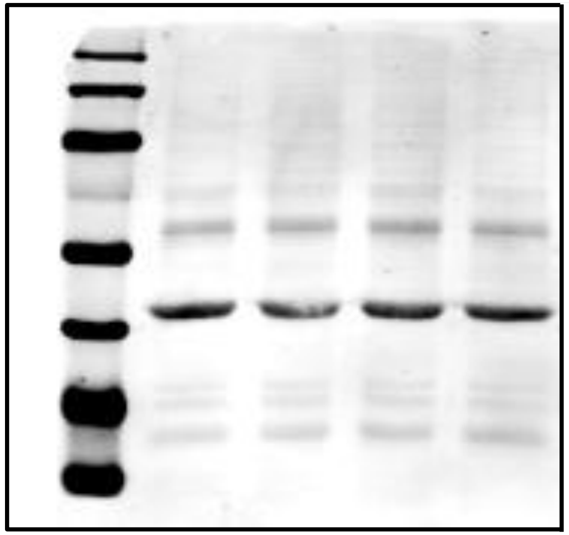

β-Actin
(predicted: 43 kd)

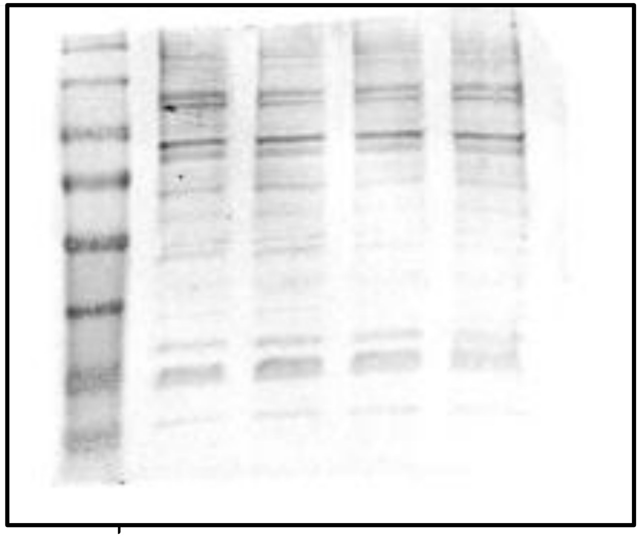

Nuclear matrix protein
(predicted: 84 kd)

Original uncropped and unadjusted blots for STAT6 in Fig. 4E

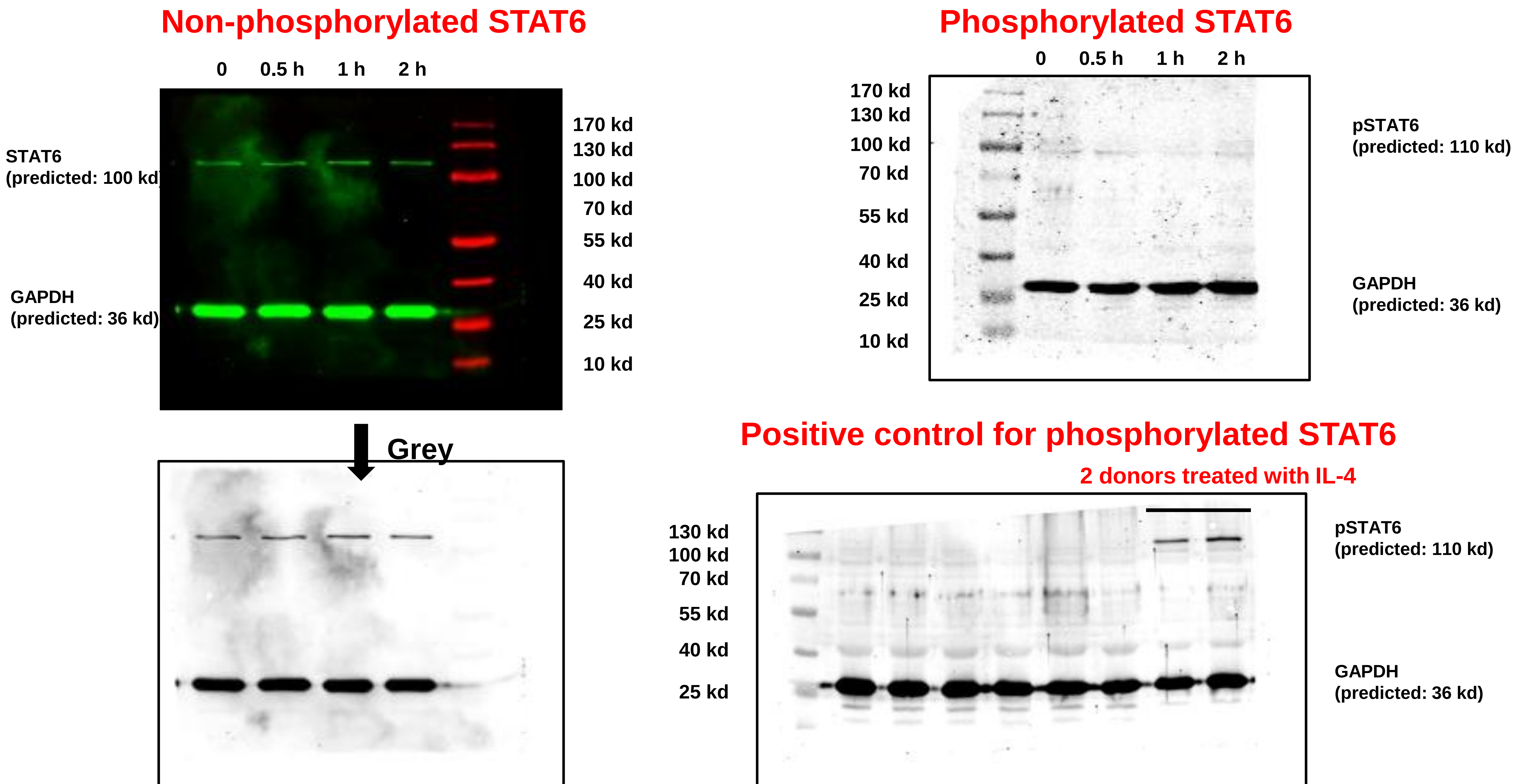

Original uncropped and unadjusted blots for COX2 in Fig. 4F

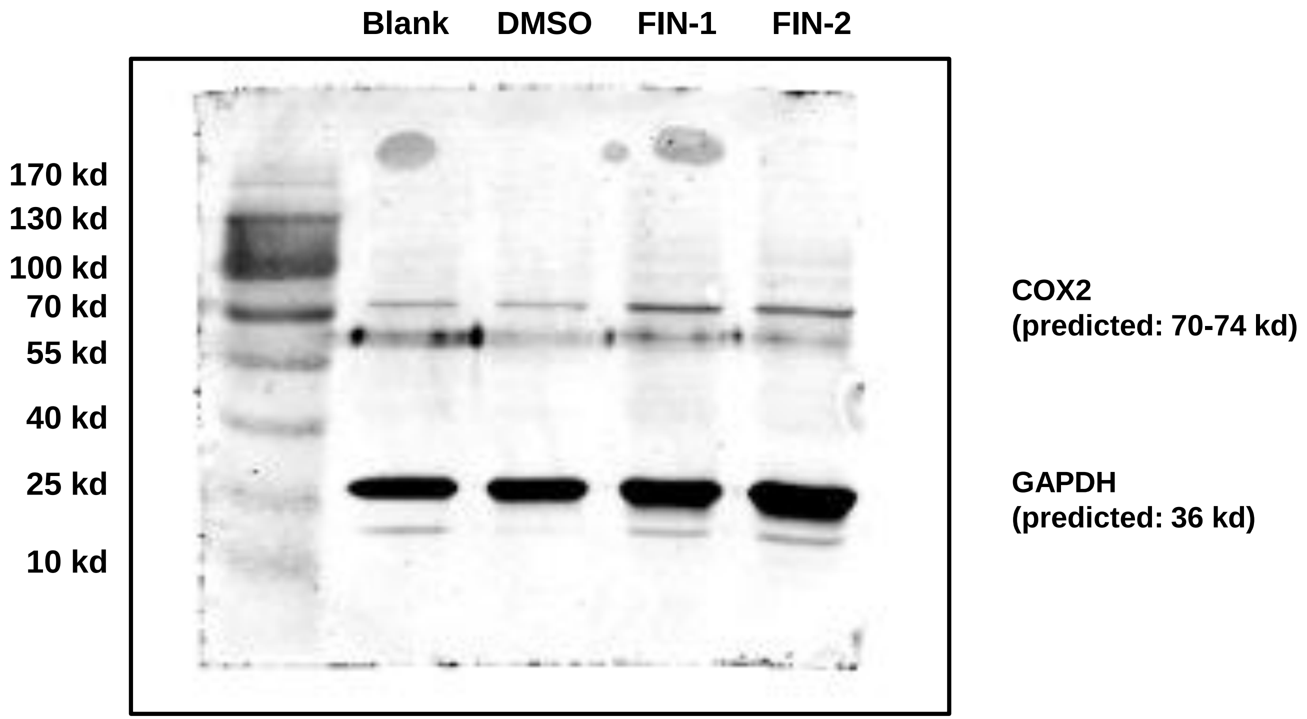

Original uncropped and unadjusted blots for COX2 in Fig. 6E

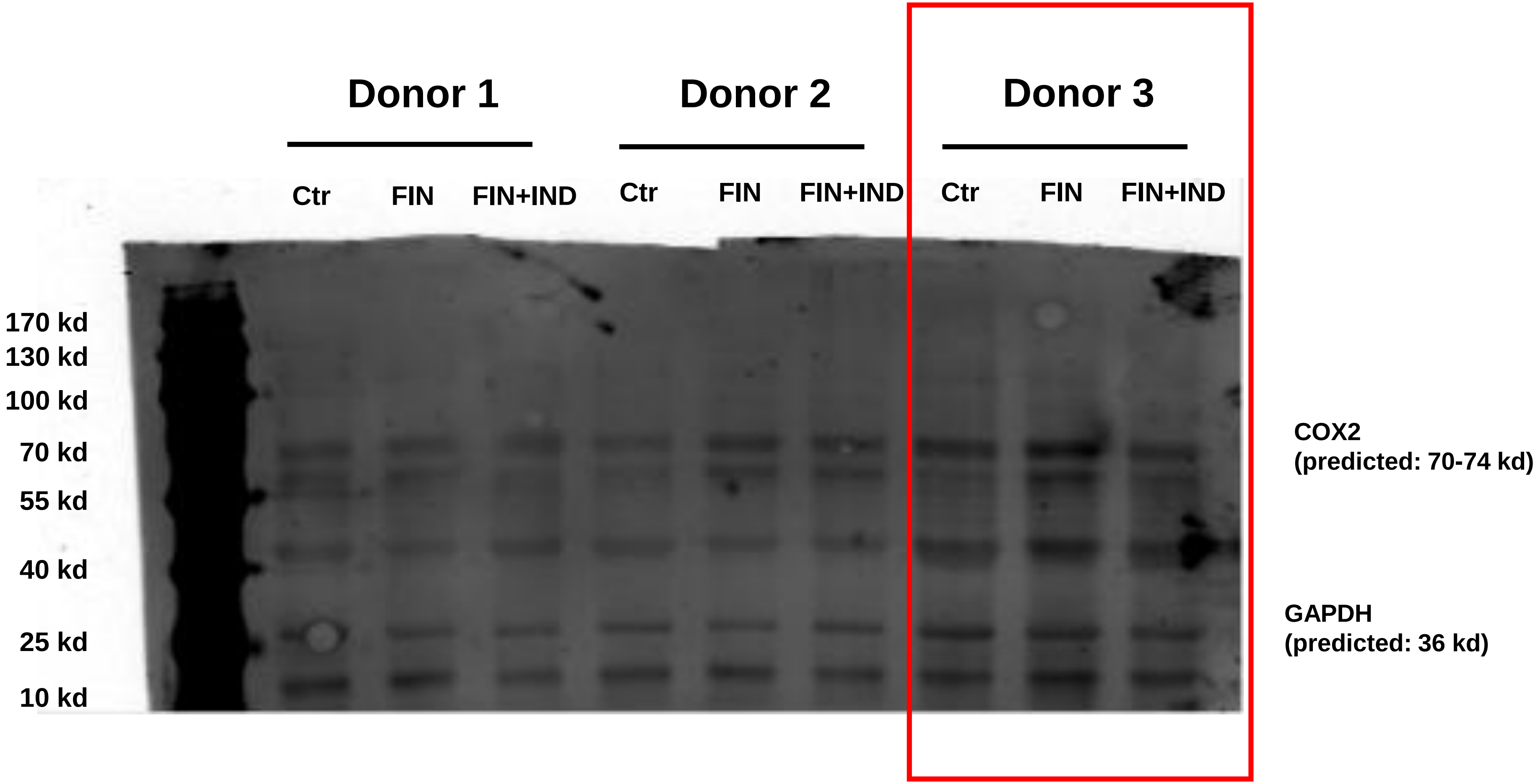

Supplement: S2 File — (PDF) [file pone.0156549.s004.pdf]
