# Supplementary material for: Finasteride Enhances the Generation of Human Myeloid-Derived Suppressor Cells by Up-Regulating the COX2/PGE2 Pathway
Source: PLoS One. 2016 Jun 2;11(6):e0156549. doi: 10.1371/journal.pone.0156549 (PMC4890941; doi:10.1371/journal.pone.0156549)
Supplement: S1 Table — (DOCX) [file pone.0156549.s005.docx]

| COX2-For | TCCTGCCTACTGGAAGCCAA |
| --- | --- |
| COX2-Rev | ATCTAGTCCGGAGCGGGAAG |
| STAT1-For | TGCTCTGAATATTCCCCGAC |
| STAT1-Rev | TTCAGGAAGACCCAATCCAG |
| STAT6-For | CAGACCCCACAGAGACATGA |
| STAT6-Rev | TCGCTGGACAGAGCTACAGA |
| NF-κB-For | GGGCCGAAAGACCTATCCC |
| NF-κB-Rev | CAGCTCCGAGCATTGCTTG |
| Arginase 1-For | TGGACAGACTAGGAATTGGCA |
| Arginase 1-Rev | CCAGTCCGTCAACATCAAAACT |
| TGFβ-For | GCAGAAGTTGGCATGGTAGC |
| TGFβ-Rev | CCCTGGACACCAACTATTGC |
| NOS2-For | TTCAGTATCACAACCTCAGCAAG |
| NOS2-Rev | TGGACCTGCAAGTTAAAATCCC |
| IFNγ-For | TGTATTGCTTTGCGTTGGAC |
| IFNγ-Rev | TGACCAGAGCATCCAAAAGA |
| IL-1β-For | AAGCCCTTGCTGTAGTGGTG |
| IL-1β-Rev | GAAGCTGATGGCCCTAAACA |
| IL-4-For | GTGTCCTTCTCATGGTGGCT |
| IL-4-Rev | CAGACATCTTTGCTGCCTCC |
| IL-13-For | CCTCATGGCGCTTTTGTTGAC |
| IL-13-Rev | TCTGGTTCTGGGTGATGTTGA |
| IL-10-For | GACTTTAAGGGTTACCTGGGTTG |
| IL-10-Rev | TCACATGCGCCTTGATGTCTG |

**S1 Table. The gene specific primers for each target genes.**
